# Molybdenum single-atoms decorated multi-channel carbon nanofibers for advanced lithium-selenium batteries

**DOI:** 10.3389/fchem.2024.1416059

**Published:** 2024-05-16

**Authors:** Yang Zheng, Mustafa Khan, Suxia Yan, Dahai Yang, Ying Chen, Li Zhang, Xiaohui Song, Guochun Li, Junfeng Liu, Yong Wang

**Affiliations:** ^1^ Institute for Energy Research, Jiangsu University, Zhenjiang, China; ^2^ School of Materials Science and Engineering, Hefei University of Technology, Hefei, China

**Keywords:** Li-Se batteries, single-atom catalyst, multi-channel carbon nanofibers, electrospun, reaction kinetics

## Abstract

The cathode in lithium-selenium (Li-Se) batteries has garnered extensive attention owing to its superior specific capacity and enhanced conductivity compared to sulfur. Nonetheless, the adoption and advancement of Li-Se batteries face significant challenges due to selenium’s low reactivity, substantial volume fluctuations, and the shuttle effect associated with polyselenides. Single-atom catalysts (SACs) are under the spotlight for their outstanding catalytic efficiency and optimal atomic utilization. To address the challenges of selenium’s low chemical activity and volume expansion in Li-Se batteries, through electrospun, we have developed a lotus root-inspired carbon nanofiber (CNF) material, featured internal multi-channels and anchored with molybdenum (Mo) single atoms (Mo@CNFs). Mo single atoms significantly enhance the conversion kinetics of selenium (Se), facilitating rapid formation of Li_2_Se. The internally structured multi-channel CNF serves as an effective host matrix for Se, mitigating its volume expansion during the electrochemical process. The resulting cathode, Se/Mo@CNF composite, exhibits a high discharge specific capacity, superior rate performance, and impressive cycle stability in Li-Se batteries. After 500 cycles at a current density of 1 C, it maintains a capacity retention rate of 82% and nearly 100% coulombic efficiency (CE). This research offers a new avenue for the application of single-atom materials in enhancing advanced Li-Se battery performance.

## 1 Introduction

As the depletion of traditional fossil fuel resources accelerates, the urgency to develop alternative energy sources becomes increasingly critical. Despite the potential of renewable energy sources such as wind power, (Ackermann and Söder, 2000), solar power, (Bharti et al., 2022), and tidal energy, (Segura et al., 2017), their applicability is hampered by inherent limitations related to time and geographical factors, posing challenges to their convenience and reliability. The rapidly evolving new energy vehicle industry has also heightened the demand for energy storage components, where the existing energy density of lithium-ion batteries (LIBs), approximately 280 Wh kg^−1^, is insufficient to satisfy the escalating energy requirements. (Xu et al., 2023).

Sulfur, with its high specific capacity of 1675 mAh g^−1^, has garnered significant interest in the field of battery technology. Nevertheless, the intrinsic poor conductivity of sulfur and the pronounced shuttle effect substantially hinder the practicality of lithium-sulfur (Li-S) batteries. (Yan et al., 2022). In search of viable alternatives, attention has shifted towards selenium (Se), a congener of sulfur in the periodic table. Lithium-selenium (Li-Se) batteries share similar advantages with their lithium-sulfur counterparts, including a high volumetric capacity of 3253 mAh cm^−3^ and a theoretical specific mass capacity of 675 mAh g^−1^. (Um et al., 2022). Furthermore, compared to S, Se exhibits higher electrical conductivity, with a value of 1 × 10^–5^ S cm^−1^ for Se and only 5 × 10^–30^ S cm^−1^ for S. (Balakumar and Kalaiselvi, 2017). The superior conductivity of Se compared to sulfur potentially enhances the reaction kinetics of Li-Se batteries. (Zeng et al., 2017). Yet, Li-Se batteries confront their own set of challenges. The electrochemical reaction in these batteries, Se + 2Li^+^ + 2e^–^ ⇆ Li_2_Se, (Kundu et al., 2013), is accompanied by significant volume changes (*i.e.*, theoretical volume expansion rate: 221.5%) and the formation of polyselenides. Such severe volume expansion can damage the electrode structure, while the soluble polyselenides lead to a shuttle effect, ultimately compromising the battery’s capacity. Additionally, the charge-discharge kinetics in Li-Se batteries are notably slow, posing substantial obstacles to their widespread adoption. (Jin et al., 2017).

Recent advancements have highlighted the efficacy of employing porous carbon as the cathode material in Li-Se batteries. (Zhang et al., 2014; Hu et al., 2021). Porous carbon, known for its excellent conductivity, offers a partial remedy to the inherently slow kinetics of Se. Encapsulating Se within porous carbon structures has proven to mitigate issues of volume expansion and the shuttle effect, prevalent in Li-Se batteries. (Balakumar and Kalaiselvi, 2017). Additionally, the selection of an appropriate electrolyte can further diminish the shuttle effect, thanks to the low solubility of polyselenides in carbonate electrolytes. (Lu et al., 2022). This low solubility promotes the discharge reaction between Se and Li metal, leading to the formation of Li_2_Se directly through a solid-to-solid reaction, bypassing any intermediates. (Abouimrane et al., 2012; Cui et al., 2014). However, such solid-to-solid reaction dynamics are inherently slow, which contributes to the observed limitations in capacity and rate performance when utilizing carbonate-based electrolytes in Li-Se batteries. (Eftekhari, 2017).

To combat the slow kinetics prevalent in Li-Se batteries, the adoption of high-performance catalysts has been identified as a promising strategy to enhance their internal chemical reactions, thereby boosting rate performance and cycling stability. Among these, single-atom catalysts (SACs) have emerged as a focal point of interest due to their distinct catalytic behaviors and superior atomic efficiency. (Cheng et al., 2022; Xing et al., 2022). SACs are characterized by their structure, consisting of individual metal atoms dispersed or anchored on a support material, offering high catalytic activity and selectivity. (Li et al., 2022; Zeng et al., 2023). The effectiveness of single-atom catalysts in Li-Se batteries has been substantiated through various studies. For instance, Tian et al., 2020 showcased the utilization of cobalt single-atom/nitrogen-doped hollow porous carbon (CoSA-HC) as a pioneering example, revealing that SACs could significantly enhance the rate capability and long-term cycling performance of Li-Se batteries. Similarly, Li and colleagues (Li et al., 2023) introduced a nickel single-atom/nitrogen-doped porous carbon nanosheet (Ni-NC) catalyst, serving as an optimal host for Se cathodes. This Ni-NC/Se cathode demonstrated a capacity of 495 mAh g^−1^ at 0.2 C, 311 mAh g^−1^ at 4 C, and sustained robust cycling stability, maintaining a capacity of 225 mAh g^−1^ after 1000 cycles at 4 C, thus highlighting the potential of SACs in improving the electrochemical performance of Li-Se batteries.

Inspired by previous researches, we developed an efficient Se host for Li-Se batteries, employing a unique structure of hollow carbon nanofibers anchored with Mo single-atom catalysts. During the electrospun solution preparation, acetylacetone molybdenum as the Mo precursor was introduced simultaneously, embedding Mo atoms within the carbon matrix uniformly. Extensive characterization confirmed the presence of numerous Mo single-atom catalytic sites within the material. Incorporating this novel material into a battery led to a substantial reduction in internal resistance, achieving a notable specific capacity of 560 mAh g^−1^ and demonstrating remarkable cycling stability at a 1 C discharge rate. Furthermore, at a higher rate of 5 C, the battery maintained a specific capacity of 490 mAh g^−1^, with capacity retention exceeding 90% even after 1000 cycles. This performance underscores the significant advantages of using Mo single-atom catalysts within multi-channel carbon nanofiber structures for enhancing the electrochemical properties of Li-Se batteries.

## 2 Experimental section

### 2.1 Reagents

Polyacrylonitrile (PAN, M_w_ = 150,000), polymethyl methacrylate (PMMA, M_w_ = 350,000), Se powder (200 mesh), and 1-Methyl-2-pyrrolidinone were supplied by Sigma-Aldrich. Anhydrous *N, N*-dimethylformamide (DMF, >99.9%) was supplied by Sinopharm Chemical Reagent Co., Ltd. Argon (Ar, 99.999%) was obtained from Zhenjiang Zhongpu Special Gas Co., Ltd. All chemical reagents were used as received, without further purification.

### 2.2 Preparation of CNFs and Mo@CNFs

Firstly, 1 g of PAN was dissolved in 6 mL of DMF and stirred at 60°C for 2 h. Subsequently, 0.4 g of PMMA was dissolved in 4 mL of DMF and heated while stirring at 60°C for 2 h. The solutions of dissolved PAN and PMMA were then mixed. After ensuring the mixture was homogenous, it was stirred continuously at 60°C for 12 h to prepare the electrospun solution. The solution was then loaded into a 10 mL syringe, extruded at a rate of 1 mL per hour, with a needle-to-receiver distance set at 15 cm, and an applied voltage of 17 kV. Following the electrospinning process, the fibers collected on the receiver were detached and dried at 60°C for 12 h to eliminate excess moisture and solvent. These fibers were then placed in a tubular furnace and pre-oxidized at 300°C in air for 3 h, with a heating rate of 5°C min^−1^. Subsequently, the pre-oxidized fibers were heated to 800°C in a tubular furnace at a heating rate of 5°C min^−1^ and maintained at this temperature for 4 h under an argon atmosphere to create the hollow structure through the thermal decomposition of PMMA. For the synthesis of Mo@CNFs, 0.4 g of PMMA and 0.16 g of molybdenum acetylacetonate were co-dissolved in 4 mL of DMF, following the same process as for the preparation of CNFs.

### 2.3 Preparation of Se/CNFs and Se/Mo@CNFs

The prepared CNFs and Se powder were combined in a mass ratio of 1:2. After thorough grinding, the mixture was placed in a tube furnace. The environment within the furnace was maintained under an argon atmosphere. The temperature was then gradually increased to 300°C at a heating rate of 5°C min^−1^ and sustained at this level for 4 h. Following this step, the gas flow rate within the tube furnace was elevated, and the temperature was further increased to 400°C, where it was maintained for 1 h. The final step aims to remove the unstable selenium from the surface of the carbon matrix. The procedure for preparing Se/Mo@CNFs followed the same methodology.

### 2.4 Physical characterization

The crystallinity and phase of the samples were analyzed using X-ray diffraction (XRD, German Bruker D8 with Cu Kα). The morphology of the carbon fibers was examined through scanning electron microscopy (SEM, JSM-7800) and transmission electron microscopy (TEM, JEM-2100 F). The selenium content was determined using thermogravimetric analysis (TGA, STA 449 F5) within a temperature range of 30°C–900°C, with a minimum heating rate of 10°C per minute. X-ray photoelectron spectroscopy (XPS, Thermo Scientific) was employed to identify the types and chemical states of the elements present. Furthermore, the specific surface area and pore size distribution, both before and after Se incorporation, were characterized using an automatic surface analyzer and a pore size analyzer, based on the Brunauer-Emmett-Teller (BET, Micromeritic ASAP 2460) method.

### 2.5 Battery fabrication

We thoroughly ground the prepared Se/Mo@CNFs and mixed Se/Mo@CNFs, Super P, PVDF in a ratio of 7:2:1, adding an appropriate amount of N-methylpyrrolidone (NMP) as a solvent. The prepared slurry was evenly coated on aluminum foil with a 200-μm scraper. The coated foil was dried overnight at 60°C in a vacuum oven, and then the foil was made into electrode slices with a diameter of 12 mm. In each electrode slice, the selenium loading amount was 0.85 ± 0.02 mg. In a glove box (H_2_O and O_2_ < 0.01 ppm), button type cells were made using CR2032 cases and Celgard polypropylene film (2400) with a diameter of 16 mm as the separator. Each battery was added with 40–45 µL of electrolyte (1 M LiPF_6_ in EC and DMC (v/v = 1:1)).

### 2.6 Electrochemical testing

The constant current charge-discharge profiles were evaluated using a Neware battery tester, with the voltage window set between 0.5 and 3.0 V. Cyclic voltammetry (CV) and electrochemical impedance spectroscopy (EIS) analyses were conducted on an electrochemical workstation (IVIMnSTAT). The CV tests covered a voltage range of 0.5–3.0 V, with scan rates of 0.1, 0.2, 0.5, 0.8, and 1 mV s^−1^. For EIS, the open circuit potential was first determined in a state of open circuit, followed by EIS measurements at this potential. The frequency range for the EIS tests was chosen to be from 0.01 Hz to 10^2^ kHz.

## 3 Results and discussions

### 3.1 Material characterization

**FIGURE 1:**
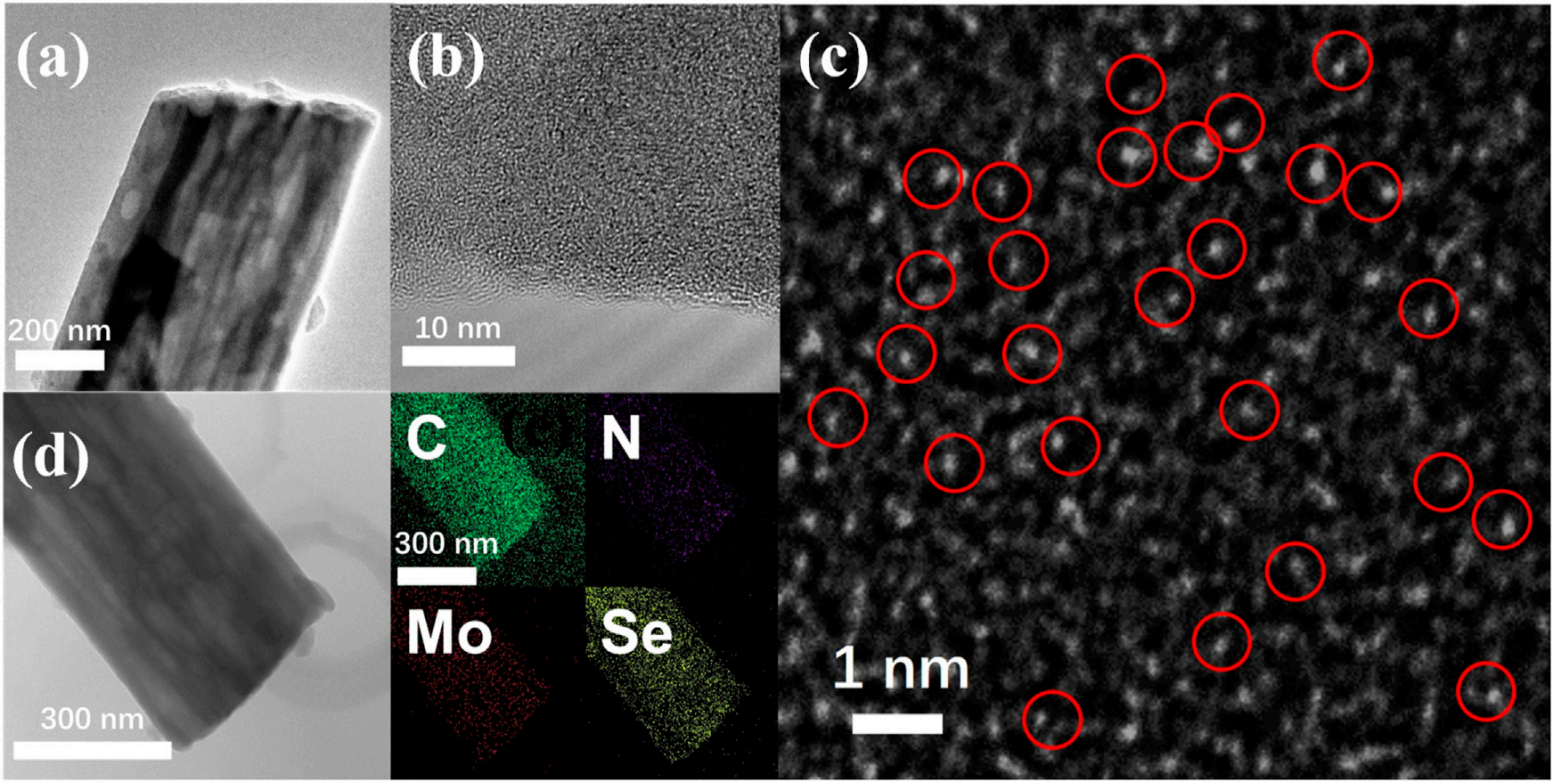
**(A)** Low and **(B)** high-magnification TEM images of Mo@CNFs, showing the detailed structure. **(C)** HAADF-STEM image of Mo@CNFs, highlighting atomic distribution of Mo element. **(D)** EDS mapping illustrating the distribution of carbon (C), molybdenum (Mo), nitrogen (N), and selenium (Se) elements in Se/Mo@CNFs.

The SEM images depicted in Supplementary Figures 1A,B illustrate the Mo@CNFs obtained through high-temperature annealing of electrospun fibers, approximately 400 nm in diameter, under an Ar atmosphere. High-resolution SEM imaging confirms that these Mo@CNFs maintain a uniform diameter akin to the original electrospun fibers, while exhibiting an internally porous structure, resulting from the decomposition of PMMA domains within the electrospun fibers. TEM analysis reveals the presence of distinct internal channels within these fibers, as illustrated in Figure 1A. Moreover, Figure 1B presents a high-magnification TEM image detailing the edge of Mo@CNFs. The image reveals that the carbon material lacks distinct lattice fringes, suggesting that the Mo@CNFs may comprise amorphous carbon. High-angle annular dark-field scanning TEM (HAADF-STEM) analysis reveals the absence of significant Mo particle agglomeration within these Mo@CNFs. Instead, numerous white dots, highlighted by red circles, are evenly dispersed across a vast carbon area (Figure 1C). This observation suggests that Mo is uniformly distributed at the atomic level within the Mo@CNFs, a finding corroborated by EDS analysis (Supplementary Figure S2), which indicates a Mo content of 5.94 wt% in the composite material (Supplementary Table S1). Figure 1D illustrates the TEM image of Mo@CNFs infused with Se (Se/Mo@CNFs), maintaining the fibrous morphology observed in Figure 1A for pristine Mo@CNFs. This consistency suggests that the Se infusion preserves the structural integrity of the hollow carbon nanofibers. Further EDS analysis highlights the uniform distribution of carbon (C), nitrogen (N), and molybdenum (Mo) elements throughout the composite, alongside a substantial and even dispersion of Se.

**FIGURE 2:**
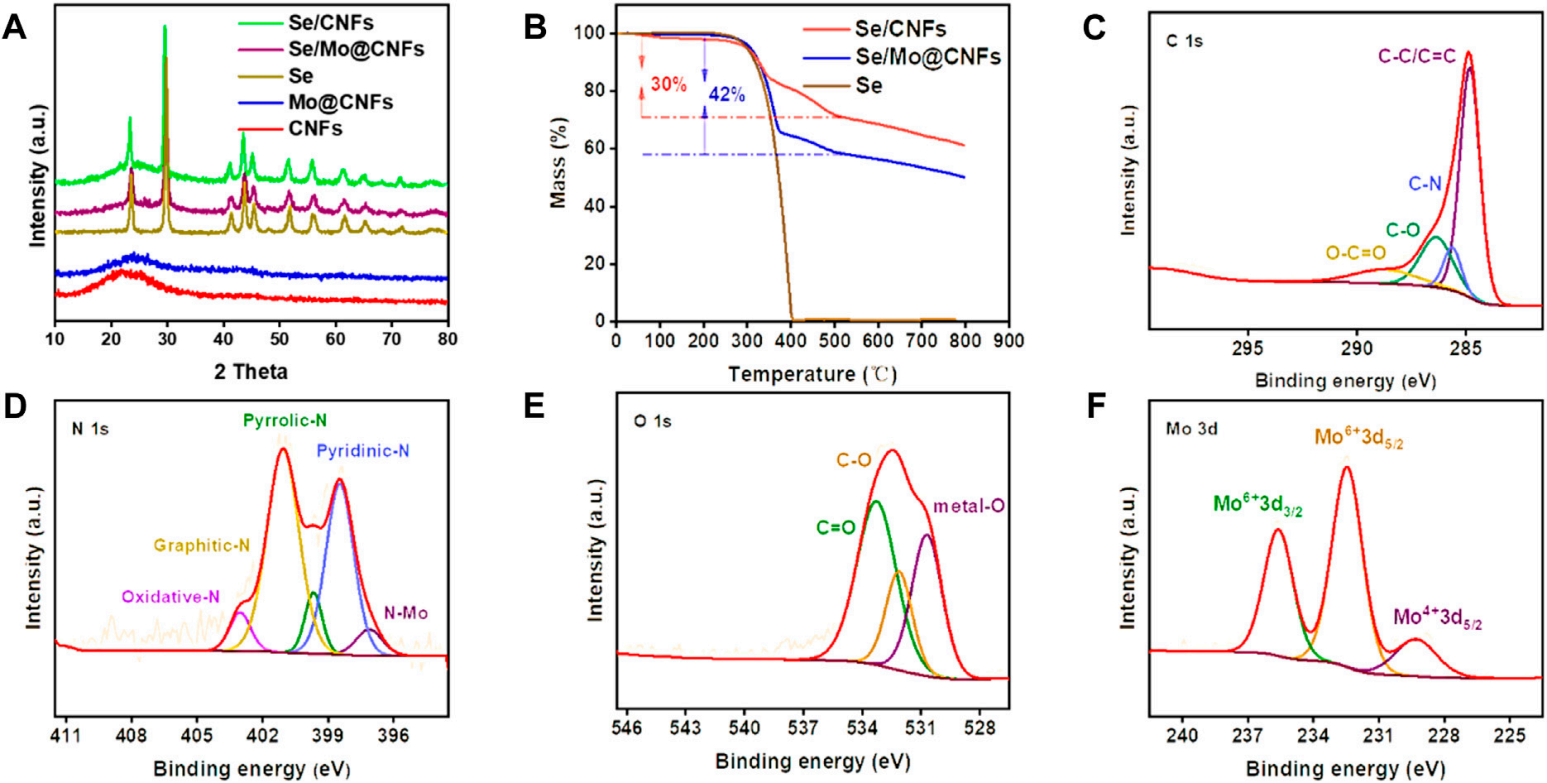
**(A)** XRD patterns of Se/CNFs, Se/Mo@CNFs, Se, Mo@CNFs, and CNFs. **(B)** TGA of Se, Se/CNFs, and Se/Mo@CNFs. **(C–F)** High resolution C 1s, N 1s, O 1s and Mo 3d XPS spectrum analysis of Mo@CNFs.

Figure 2A displays the XRD patterns for CNFs, Se/CNFs, Mo@CNFs, and Se/Mo@CNFs. The patterns for CNFs and Mo@CNFs exhibit the characteristic peaks of carbon materials, with a distinct broad peak at approximately 24° corresponding to amorphous carbon. The absence of graphite’s (100) and (101) peaks in XRD indicates that the carbon in CNFs lacks three-dimensional ordered stacking. (Kim et al., 2007; Lai and Lo, 2015). Previous studies have reported that in PAN/PMMA mixed carbon fibers, the continuous release of heteroatoms due to PMMA decomposition leads to the formation of a disordered layered structure, resulting in a transition from ordered graphite to amorphous carbon along with the amount of PMMA increasing. (Li et al., 2018). In our CNFs electronspun solution, the PAN:PMMA ratio is 10:4, with a substantial amount of PMMA doping contributing to lower degree of graphitization of the material, primarily consisting of amorphous carbon.

Notably, the absence of diffraction peaks for Mo in Mo@CNFs suggests atomic dispersion of Mo within the structure. The XRD patterns of Se/Mo@CNFs composite, in contrast, show sharp and intense peaks aligning with those of pure Se, indicating the presence of Se on the surface of the composite. Se incorporation into the matrix material was achieved using the melt diffusion method. To demonstrate the effectively accommodation of Se, we determined the BET specific surface area and pore structure of Mo@CNFs through nitrogen adsorption/desorption isotherm analysis. As shown in Supplementary Figure S3A, both Mo@CNFs and Se/Mo@CNFs exhibit Type IV curves with H1 hysteresis loops, which is a typical characteristic of mesoporous materials. Its pore size is mainly distributed at 37 nm (Supplementary Figure S3B). The specific surface area of Mo@CNFs is 246 m^2^ g^−1^, whereas after Se infusion, the specific surface area of Se/Mo@CNFs decreases to 15 m^2^ g^−1^, indicating complete Se filling of the pores in Mo@CNFs. Thermogravimetric analysis (TGA) depicted in Figure 2B reveals a two-stage weight loss for Se/Mo@CNFs. The initial stage, ranging from 300°C to 380°C, corresponds to the volatilization of surface Se, similar to the weight loss pattern of pure Se. A subsequent weight loss phase observed above 380°C is attributed to the volatilization of Se residing within the mesopores of the material. (Liu et al., 2017). This observation indicates that the Se loading in the Se/Mo@CNFs composite is approximately 42% by weight, whereas in Se/CNFs, the loading is around 30%.

The composition analysis of Mo@CNFs, specifically the C, O, N, and Mo elements, was elucidated through X-ray photoelectron spectroscopy (XPS) characterization. The survey spectra demonstrate that Mo@CNFs consist primarily of elements such as C, N, O, and Mo. Carbon originates mainly from the thermal decomposition of PAN and PMMA, (He et al., 2018), while N is derived from PAN, and O primarily arises from the dehydrogenation and pre-oxidation process of the fibers. As illustrated in Figure 2C, the high-resolution C 1s spectrum of Mo@CNFs exhibits four prominent peaks: C=O (288.8 eV), C-O (286.4 eV), C-N (285.7 eV), and C-C/C=C (284.8 eV), with the C-C/C=C peak being the most pronounced, indicating a partial transformation to amorphous carbon. (Li F. et al., 2021). The high-resolution N 1s spectrum of Mo@CNFs, as shown in Figure 2D, displays peaks at 398.4, 399.8, 401.1, 403.3 eV, and 396.2 eV corresponding to pyridinic-N, pyrrolic-N, graphitic-N, oxidized-N, and N-Mo respectively. (Guo et al., 2016). This variety of nitrogen functionalities enhances the conductivity of carbon-based materials and accelerates electrochemical reactions. (Eng et al., 2021). The discovery of N-Mo indicates the coordination of N with Mo to form Mo-N_x_ sites, which are integrated into the carbon matrix. (Ma et al., 2020). The high-resolution O 1s spectrum of Mo@CNFs (Figure 2E) can be analyzed by deconvolution into 533.5 eV, 532.1 eV, 530.7 eV. The peaks at 533.5 eV and 532.1 eV correspond to C-O and C=O, respectively. These oxygen species are primarily derived from the pre-oxidation process of PAN. The peak at 530.7 eV can be attributed to metal-O interaction, indicating the presence of Mo combined with O within the material. (Geng et al., 2021).

Analysis of the Mo spectra reveals peaks at 235.6, 232.5, and 229.3 eV (Figure 2F), with 232.5 eV corresponding to the 3d_3/2_ of Mo^4+^ and 235.6, 229.3 eV to the 3d_5/2_ of Mo^6+^, (Li TF. et al., 2021; Kim et al., 2021), indicating that Mo primarily exists in the single-atom forms of Mo^4+^ and Mo^6+^. (Zhang et al., 2019). Upon diffusion of Se into Mo@CNFs to form the Se/Mo@CNFs compoiste, notable changes are observed in the high-resolution Mo 3d spectrum (Supplementary Figure S4B). Specifically, a prominent shift is evident at 238.0 eV, indicative of the formation of Mo-Se bonds within the composite material. (Liu et al., 2015). Additionally, upon examining the high-resolution Se 3d spectrum (Supplementary Figure S5) of the Se/Mo@CNFs composite, four distinct peaks are identified. Peaks at 59.2 eV and 58.1 eV correspond to Se 3d_3/2_ and Se 3d_5/2_, respectively, typically suggesting the presence of Se-O and Se-C bonds. Furthermore, the peaks at 56.4 eV (Se 3d_3/2_) and 55.5 eV (Se 3d_5/2_) align with those observed in typical Se/C composite materials. (Sha et al., 2018; Wang et al., 2018).

### 3.2 Electrochemical testing

**FIGURE 3:**
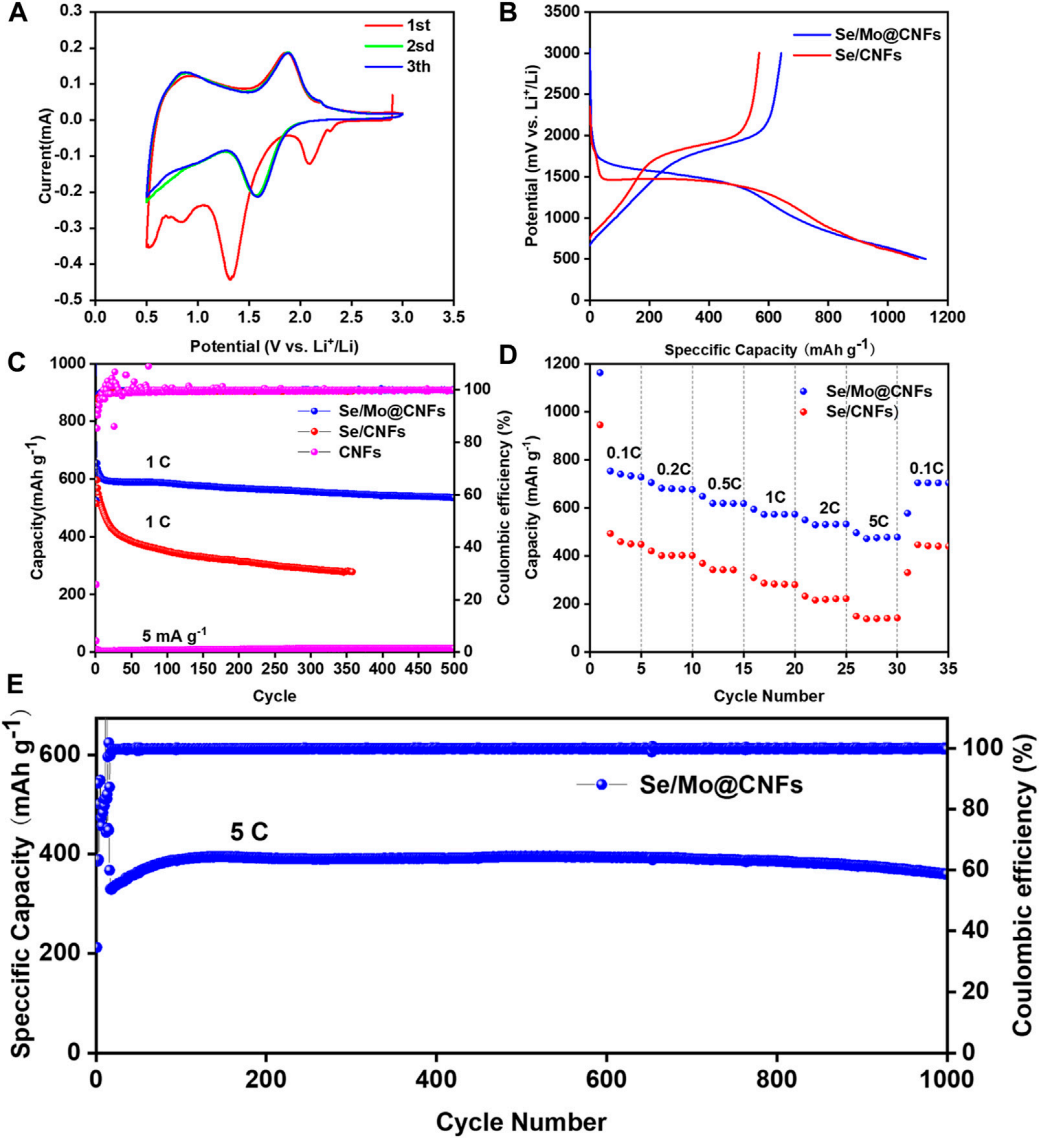
Electrochemical evaluation of CNFs, Se/CNFs, and Se/Mo@CNFs: **(A)** CV curves for Li-Se battery equipped with Se/Mo@CNFs cathode at a scan rate of 0.1 mV/s over the initial three cycles. **(B)** First-cycle discharge-charge voltage profiles for Se/CNFs and Se/Mo@CNFs cathodes at a current of 1 C rate. **(C)** Cycling performance comparison of these batteries at a 1 C rate. **(D)** Rate capability of Se/CNFs *versus* Se/Mo@CNFs cathode. **(E)** Long-term cycling stability of Se/Mo@CNFs cathode conducted at a 5 C rate.

Figure 3A presents the CV characterization of a Li-Se cell incorporating a Se/Mo@CNFs cathode over the initial three sweeps at a scan rate of 0.1 mV s^−1^ within a 0.5–3 V voltage window. Notably, an oxidation peak emerges near 2.1 V during the first discharge of the Se/Mo@CNFs cathode, which is absent in subsequent cycles. This phenomenon is attributed to the lithiation reactions of various Se species within the material. (Li et al., 2014). During this process, Se is reduced from Se_8_ to Se_n_ (n > 4), forming an amorphous state, and lithiated to Li_2_Se_n_ (n > 4), ultimately yielding Li_2_Se near 1.5 V. Moreover, the CV curve of the first discharge cycle exhibits a significant deviation from that of the second cycle. This discrepancy may be because the lithiation process during the initial discharge cycle primarily occurs on the surface of the material, where the discharge potential is directly related to the form of Se present on the material surface. (Zhou et al., 2015). This observation corresponds to the partial crystalline Se on the material’s surface observed in the XRD patterns (Figure 2A). Subsequent to the second cycle, the CV profiles of Se/Mo@CNFs show substantial overlap, indicating the material’s excellent electrochemical stability. This comparison also underscores the superior reversibility of Se/Mo@CNFs compared to Se/CNFs. (Sun et al., 2023).

Figure 3B illustrates the discharge/charge profiles of the first cycle for Se/Mo@CNFs and Se/CNFs at a rate of 1 C (where 1 C = 675 mA g^−1^). Se/Mo@CNFs exhibits distinct discharge and charge plateaus at 1.8 V and 1.5 V, respectively, consistent with its CV characteristics. The initial discharge capacity for Se/Mo@CNFs measures 1160 mAh g^−1^, with a subsequent charge capacity of 670 mAh g^−1^. The capacity loss, likely attributable to the formation of a solid electrolyte interface (SEI) layer during the initial cycle, contributes to an irreversible capacity decrease. (Luo et al., 2014). The first-cycle CE of Se/CNFs is 51.6%, contrasted with 57.0% for Se/Mo@CNFs, indicating a higher CE and Se utilization in the latter.

During extended cycling (depicted in Figure 3C), Se/Mo@CNFs maintain a capacity of 535 mAh g^−1^ after 500 cycles at a 1 C rate, demonstrating a capacity retention of 82% and an average capacity fade of 0.36% per cycle. Conversely, Se/CNFs exhibit a pronounced capacity decline under the same conditions. To further highlight the exceptional specific capacity performance of Se/Mo@CNFs, we compared recent reports on Li-Se battery cathodes (Supplementary Table S2). After 500 cycles, Se/Mo@CNFs demonstrate significantly higher specific capacity retention compared to other Li-Se battery cathodes, establishing it as an outstanding cathode material. To assess the specific contribution of Se loading, we conducted long-cycle tests on CNFs and Mo@CNFs without Se loading. At a current density of 5 mA g^−1^, both CNFs and Mo@CNFs exhibit low specific capacities of 13 mAh g^−1^ (Figure 3C) and 15 mAh g^−1^ (Supplementary Figure S6), respectively. These capacities are considerably lower compared to the capacities achieved after Se loading, indicating minimal contribution of pure CNFs and Mo@CNFs to the discharge capacity in these Li-Se batteries.

The rate performance of Se/Mo@CNFs was evaluated across various current densities ranging from 0.1 C to 5 C (Figure 3D). At these rates, the Se/Mo@CNFs cathode demonstrated discharge capacities of 727.94 mAh g^−1^ at 0.1 C, 676.04 mAh g^−1^ at 0.2 C, 617.58 mAh g^−1^ at 0.5 C, 572.93 mAh g^−1^ at 1 C, 531.96 mAh g^−1^ at 2 C, and 477.7 mAh g^−1^ at 5 C, respectively. Remarkably, when the rate was dialed back to 0.1 C, the capacity of Se/Mo@CNFs rebounded to 703.91 mAh g^−1^, showcasing superior rate performance compared to Se/CNFs. Remarkably, even at a challenging rate of 5 C, Se/Mo@CNFs exhibited minimal capacity loss, highlighting its robust resilience to high-rate discharge conditions. Following 1000 cycles at 5 C (depicted in Figure 3E), Se/Mo@CNFs maintained a capacity of 367 mAh g^−1^. This capacity exhibited a gradual increase over the initial fifty cycles, likely attributed to an activation process induced by the elevated initial discharge rate.

**FIGURE 4:**
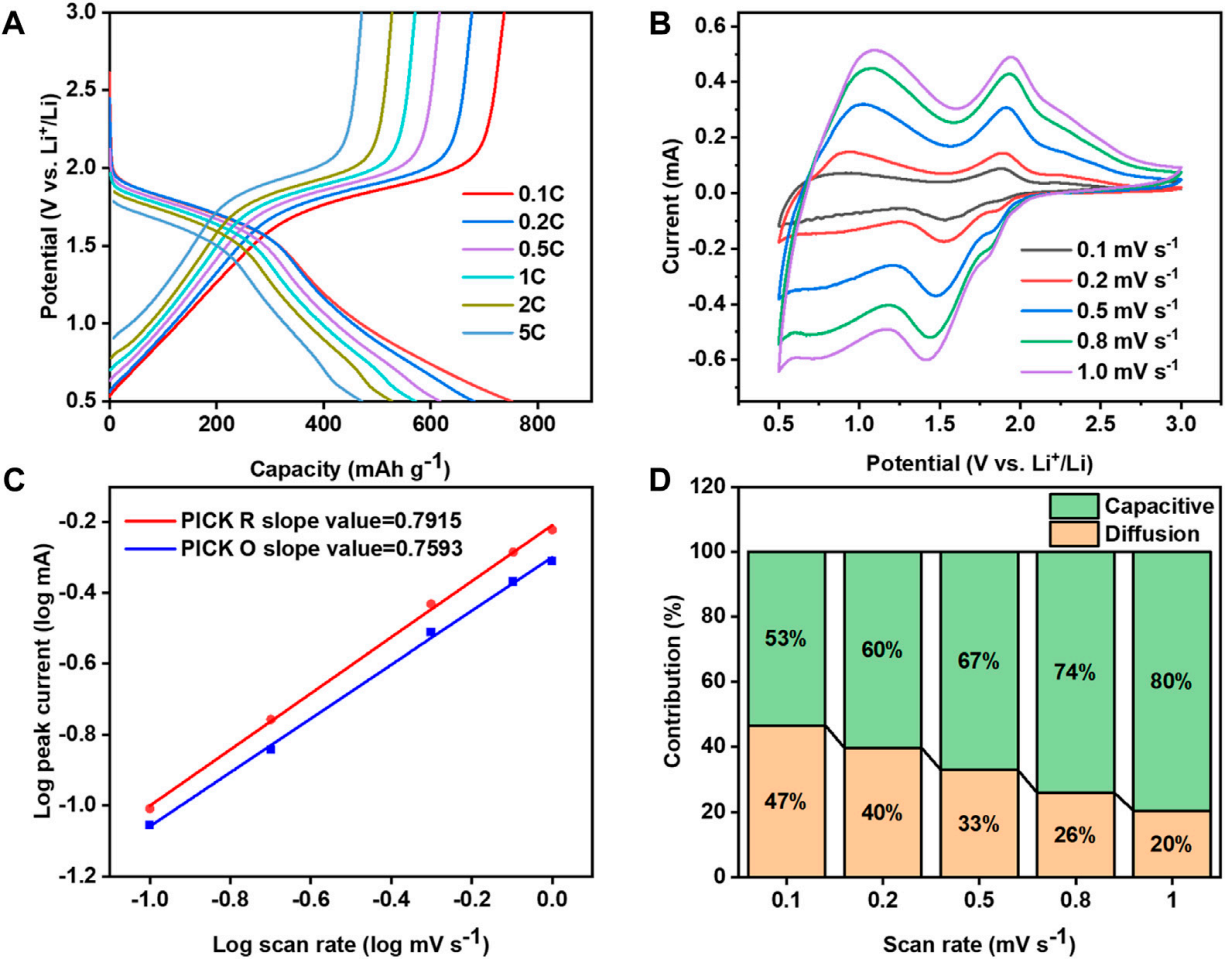
**(A)** Discharge-charge voltage profiles across a range of current densities from 0.1 to 5 C rate. **(B)** CV curves obtained at varying scan rates. **(C)** Plot of log current (log(i)) *versus* log scan rate (log(v)), illustrating the relationship. **(D)** Graph showing the normalized ratio of diffusion-controlled to capacitive contributions at different scan rates, highlighting the reaction kinetics.

The cathode of Li-Se batteries often encounters challenges related to sluggish reaction kinetics, a concern thoroughly investigated in this study. We utilized a variety of techniques to explore the Se/Mo@CNFs cathode, including charge-discharge cycling at varying current densities, CV testing across a spectrum of scan rates, and Tafel slope analysis to evaluate reaction kinetics. Figure 4A and Supplementary Figure S7 show the charging and discharging profiles of Se/Mo@CNFs and Se/CNFs at different current densities, respectively. A comparison of these two profiles reveals that Se/Mo@CNFs exhibit a distinct voltage plateau as the current density increases from 0.1 C to 5 C, while the voltage plateau for Se/CNFs becomes less pronounced during this process. Additionally, at the same current density, the potential difference between the charging and discharging profiles of Se/Mo@CNFs is smaller, indicating reduced polarization and better kinetic performance compared to Se/CNFs.

The CV scan rate was progressively increased from 0.1 mV s^−1^ to 1 mV s^−1^, and as observed in Figure 4B, the CV curve peaks of Se/Mo@CNFs cathode exhibit only minor shifts with increasing scan rates, suggesting a minimal polarization inside the battery within the battery system. (Xiao et al., 2020; Li XY. et al., 2021). In contrast, the CV scan spectra of Se/CNFs exhibited severe distortion (Supplementary Figure S8), with the peak of the CV curve moving to higher voltages as the scan rate increased. This indicates that Se/Mo@CNFs demonstrate more effective reaction kinetics compared to Se/CNFs.

To comprehensively investigate the electrochemical reaction dynamics within Li-Se batteries, we examined the correlation between the peak current ( i ) and the scan rate ( v ), as demonstrated by the following equation (Deng et al., 2018):

$$\log\;\left(i\right)=blog\;\left(v\right)+\log\;\left(a\right)$$ (1)

in Eq. (1) the parameters *a* and *b* are derived from the observation in CV testing. The value of parameter *b*, ranging from 0.5 to 1, serves as a crucial indicator. A *b* value nearing 0.5 implies a diffusion-controlled process during the electrochemical reaction, while a *b* value approaching 1 indicates an interface-controlled process with significant capacitive behavior, signifying faster kinetics. (Zhang et al., 2016) The calculated *b* values of 0.7915 and 0.7593 (Figure 4C), obtained from peak currents at various sweep rates, signify the coexistence of both diffusion and capacitive processes within the reaction dynamics. By employing the formula $i=K_{1}v+K_{2}v^{0.5}$ , we quantified the contributions of capacitive and diffusion processes to the reaction. Here, K_1_ν and K_2_ν^0.5^ represent capacitive and diffusive contributions, respectively. As illustrated in Figure 4D, with the increase in sweep rate from 0.1 mV s^-1^ to 1 mV s^−1^, the capacitive contribution escalates from 53% to 80%. This augmentation in capacitance facilitates Li^+^ transport, thereby enhancing the battery’s cycle stability and rate performance. (Chen et al., 2017)

**FIGURE 5:**
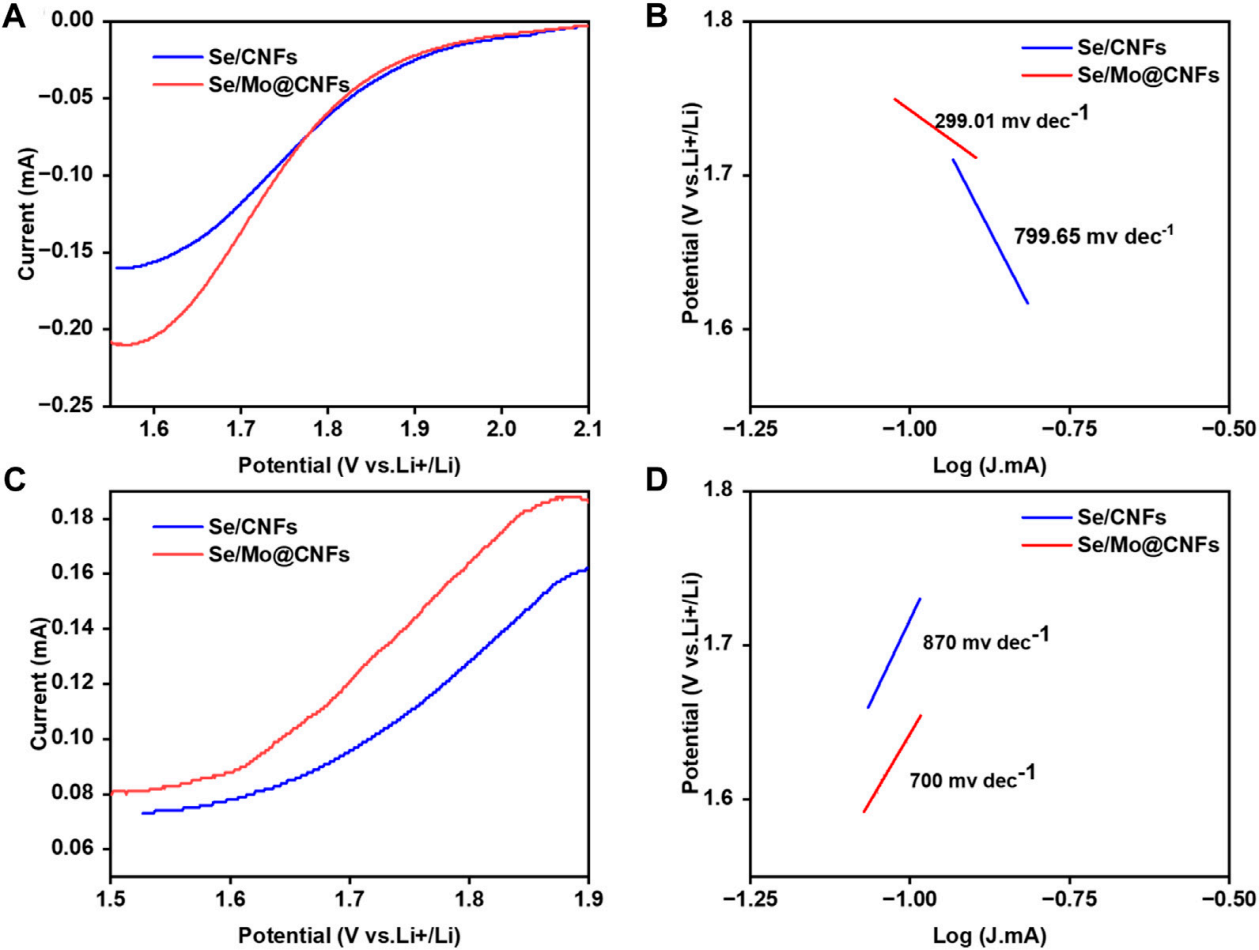
Electrochemical testing of Se/CNFs and Se/Mo@CNFs. **(A)** and **(B)** CV for the Li_2_Se reduction and Tafel plots for the Li_2_Se reduction. **(C)** and **(D)** CV for the Li_2_Se oxidation and Tafel plots for the Li_2_Se oxidation.

To visually demonstrate the influence of Mo@CNFs on the kinetic behavior of Li-Se batteries, we conducted Tafel kinetic tests on both the reduction and oxidation processes of Se in Se/Mo@CNFs and Se/CNFs cathodes separately. By comparing the Tafel slopes associated with these processes, the influence of each material on the polyselenides conversion rate is discernible. Specifically, during the Se reduction process (refer to Figure 5), the Tafel slope for Se/Mo@CNFs is 299.01 mV dec^−1^, significantly lower than Se/CNFs’ 799.65 mV dec^−1^ slope observed for Se/CNFs. This stark contrast indicates that Mo@CNFs substantially enhance the internal chemical kinetics of the battery system. (Li et al., 2019; Liu et al., 2021).

Furthermore, EIS tests unveiled that Se/Mo@CNFs manifest significantly lower impedance in contrast to Se/CNFs, indicating superior electron transport capabilities. Notably, after 100 cycles at a 1 C rate, the impedance of both Se/CNFs and Se/Mo@CNFs decreased, with Se/Mo@CNFs demonstrating a notably lower impedance than Se/CNFs after the same number of cycles (Supplementary Figure S9). This decrease in impedance further emphasizes the augmented electrochemical performance and efficiency offered by the Se/Mo@CNFs electrode material. After subjecting the cell to 1000 cycles at 5 C, we disassembled the cell and washed the Se/Mo@CNFs electrode with DMC for preparation the SEM sample of electrode after cycling. The comparison of SEM images (Supplementary Figure S10) before and after cycling reveals that Se/Mo@CNFs maintain their complete fibrous state, even after 1000 cycles at 5 C. This observation underscores the exceptional cyclic performance and high reliability of Se/Mo@CNFs as an electrode material for Li-Se batteries.

### 3.3 Conclusion

In conclusion, this study introduces and implements a novel approach by synthesizing and employing molybdenum single-atom catalysts (Mo@CNFs) embedded within hollow carbon nanofibers as a promising Se host in Li-Se batteries. The ingeniously designed multi-channel architecture within these carbon nanofibers not only accommodates Se effectively but also significantly enhances the overall battery performance in terms of cycle stability, rate capability, and CE. This enhancement is primarily attributed to the catalytic prowess of the Mo single-atom sites, which expedite the electrochemical reactions of Se. Remarkably, even after 1000 cycles at an aggressive 5 C current rate, the Se/Mo@CNFs cathode retains its structural integrity, underscoring its remarkable durability. Furthermore, comprehensive electrochemical analyses, including CV at varied sweep rates and Tafel plots, unequivocally demonstrate the exceptional role of Mo@CNFs in facilitating Se’s reaction kinetics within carbonate electrolytes. This work not only highlights the potential of single-atom catalysts in advancing Li-Se battery technology but also opens new pathways for the development high-performance energy storage systems with broader implications in sustainable energy applications.

## Data Availability

The original contributions presented in the study are included in the article/Supplementary Material, further inquiries can be directed to the corresponding authors.
