# Supplementary material for: Molybdenum single-atoms decorated multi-channel carbon nanofibers for advanced lithium-selenium batteries
Source: Front Chem. 2024 May 16;12:1416059. doi: 10.3389/fchem.2024.1416059 (PMC11141169; doi:10.3389/fchem.2024.1416059)
Supplement: Supplementary file 1 [file DataSheet1.PDF]

Supplementary Material

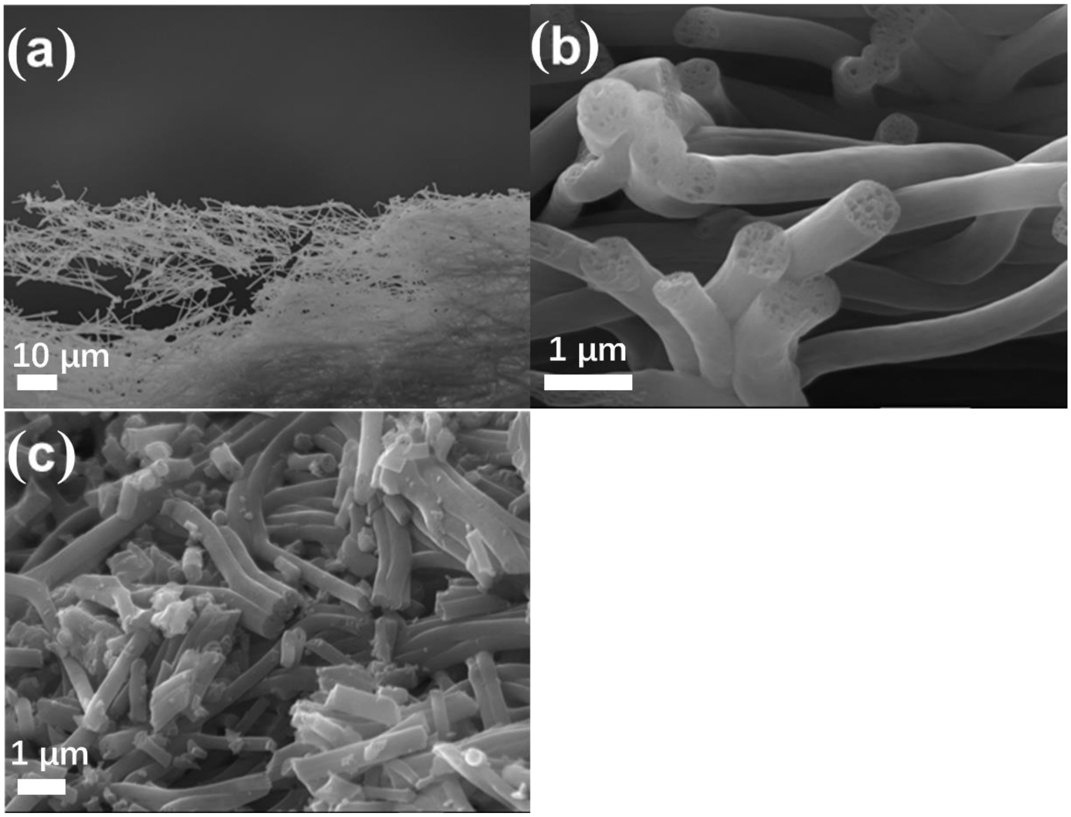

Supplementary Figure 1. (a) and (b) SEM images of Mo@CNFs at low and high magnification. (c) SEM images of Se/Mo@CNFs pole piece.

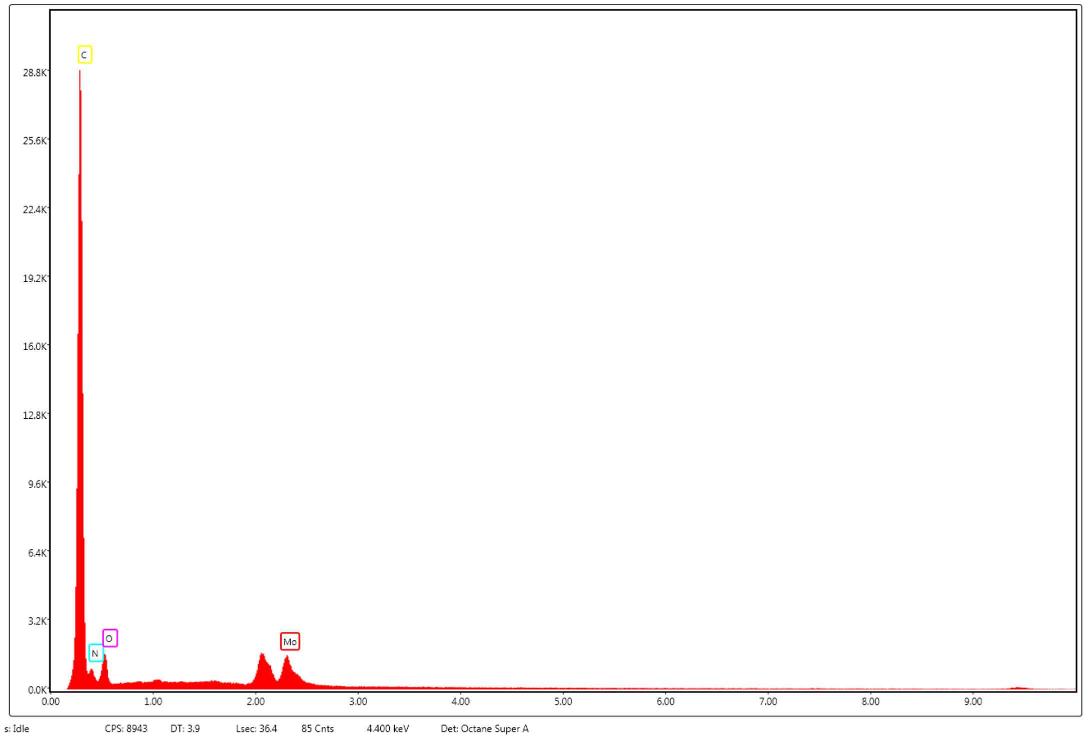

Supplementary Figure 2. The EDS spectrum of Mo@CNFs.

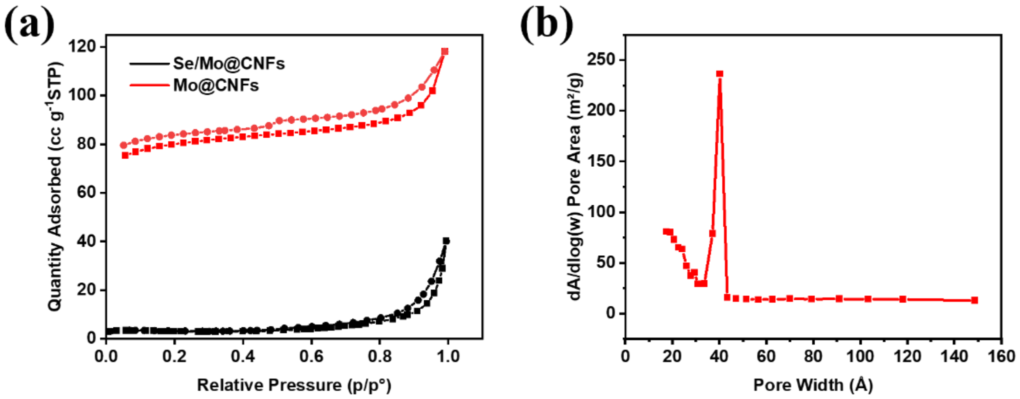

Supplementary Figure 3. (a) Adsorption-desorption isotherms of Mo@CNFs and Se/Mo@CNFs, (b) Pore size distribution of Mo@CNFs.

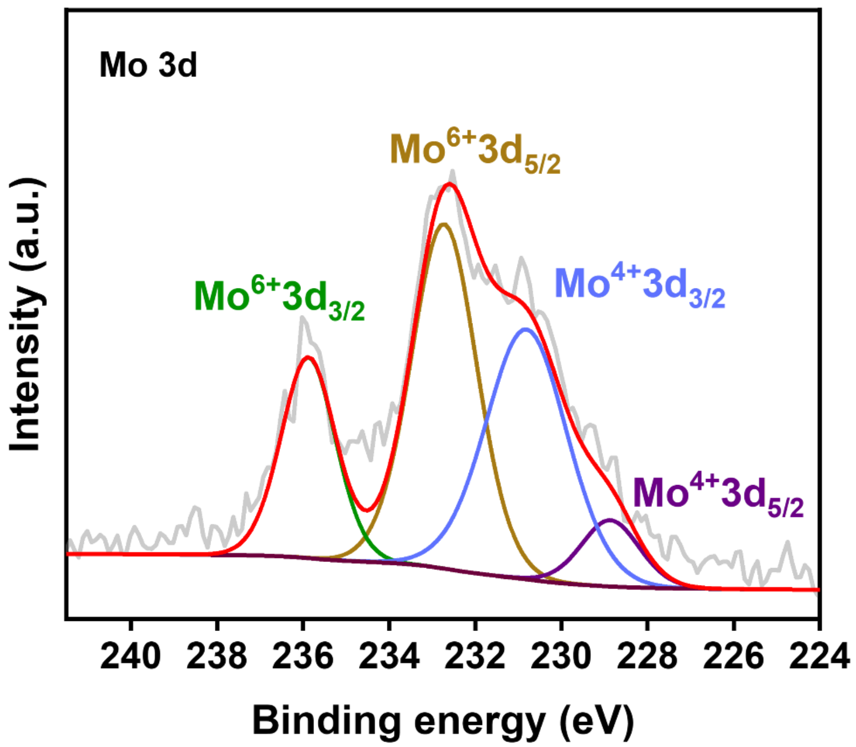

Supplementary Figure 4. Mo 3d of Se/Mo@CNF.

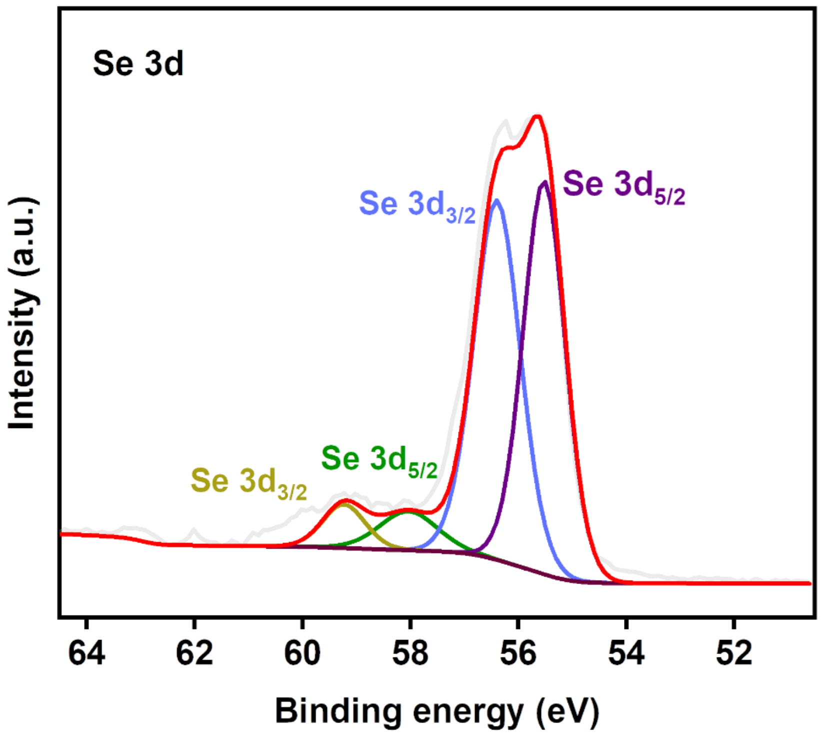

Supplementary Figure 5. Se 3d of Se/Mo@CNFs.

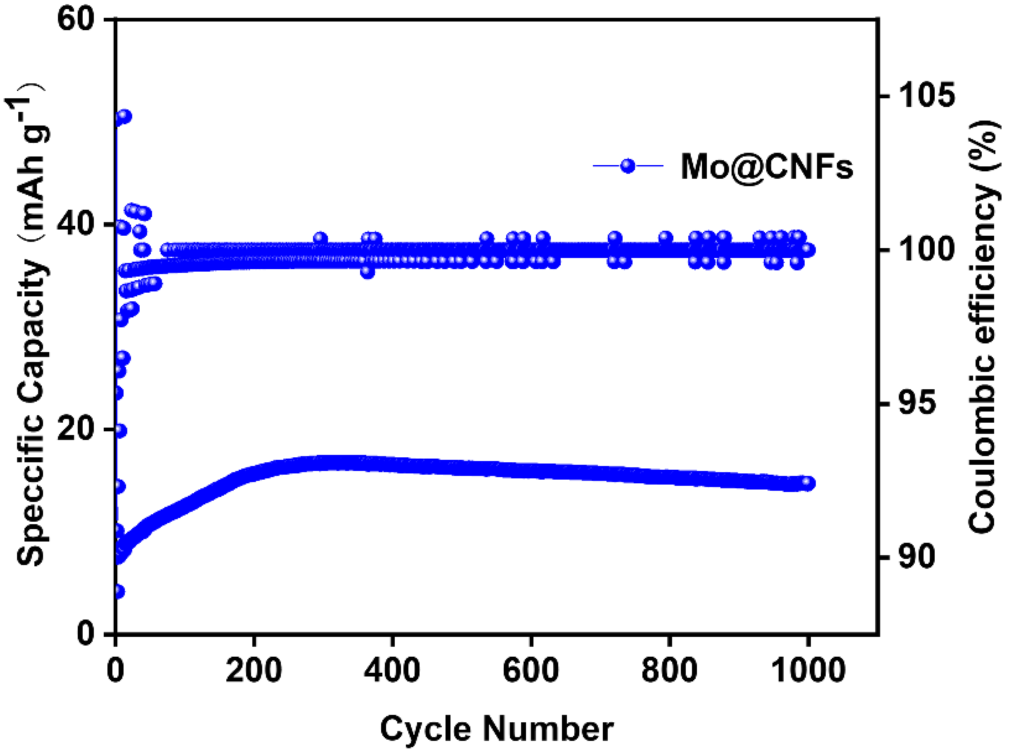

Supplementary Figure 6. The cycling performance of Mo@CNFs at a current density of 5 mA g^{-1}.

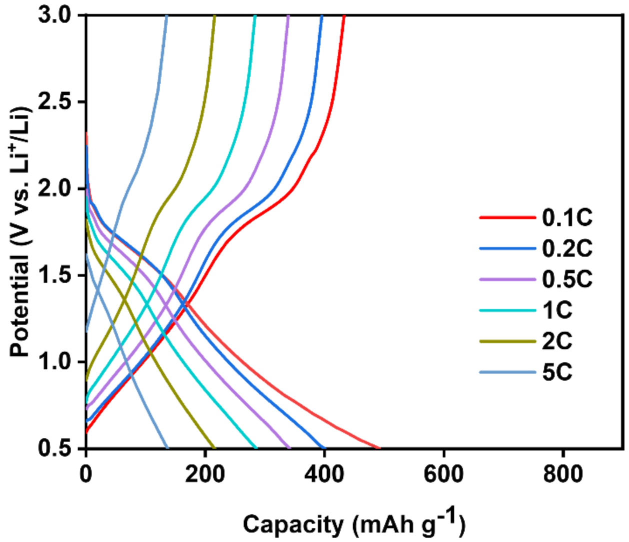

Supplementary Figure 7. Se/CNFs discharge-charge voltage profiles across a range of current rates from 0.1 C to 5 C.

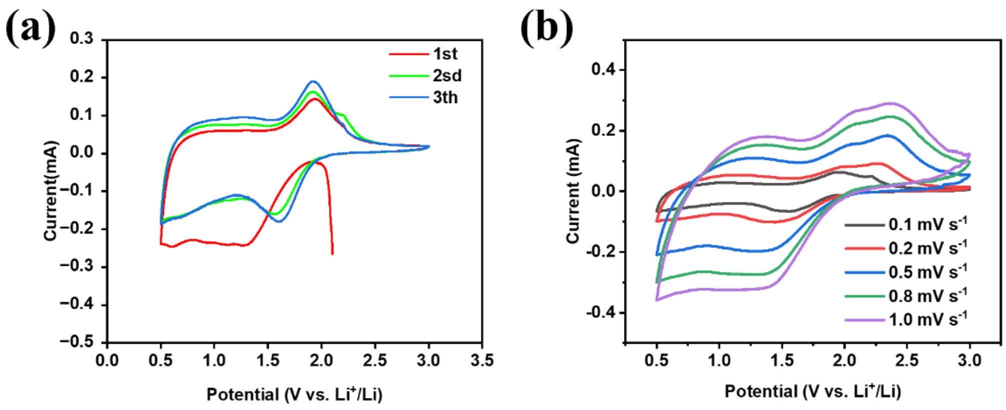

Supplementary Figure 8. (a) CV curves of Se/CNFs at a scanning rate of 0.1 mV s^{-1} (first three cycles) (b) CV curves at various scan rates of Se/CNFs.

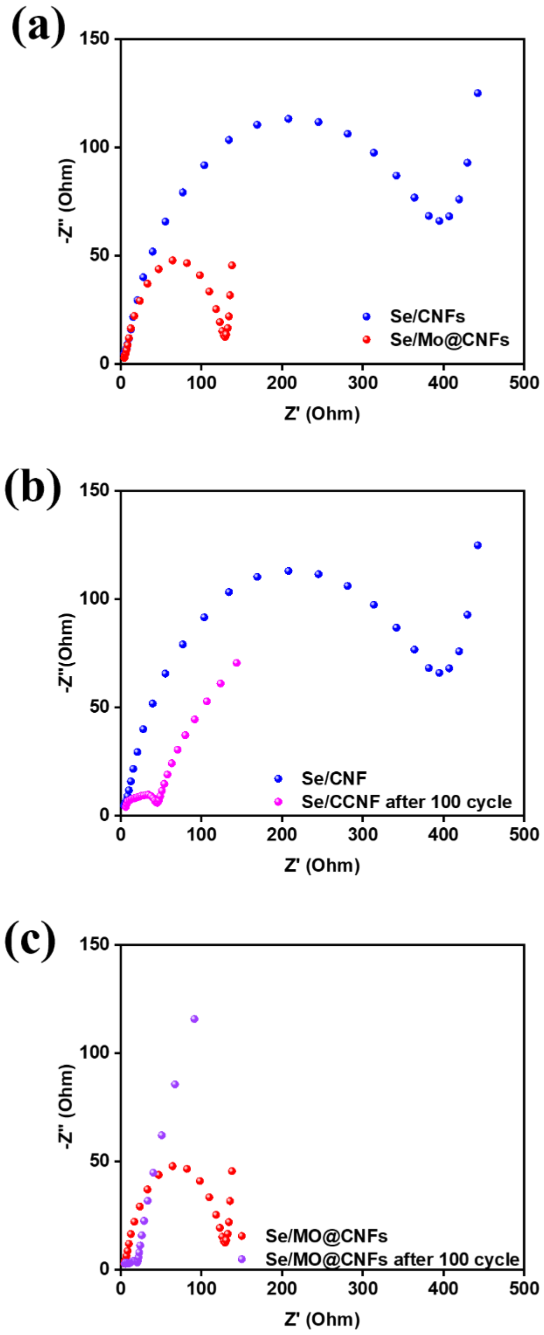

Supplementary Figure 9 (a) EIS before the cycle of Se/CNFs and Se/Mo@CNFs (b) EIS before and after 100 cycles of Se/CNFs (c) Comparison of EIS before and after 100 cycles of Se/Mo@CNF.

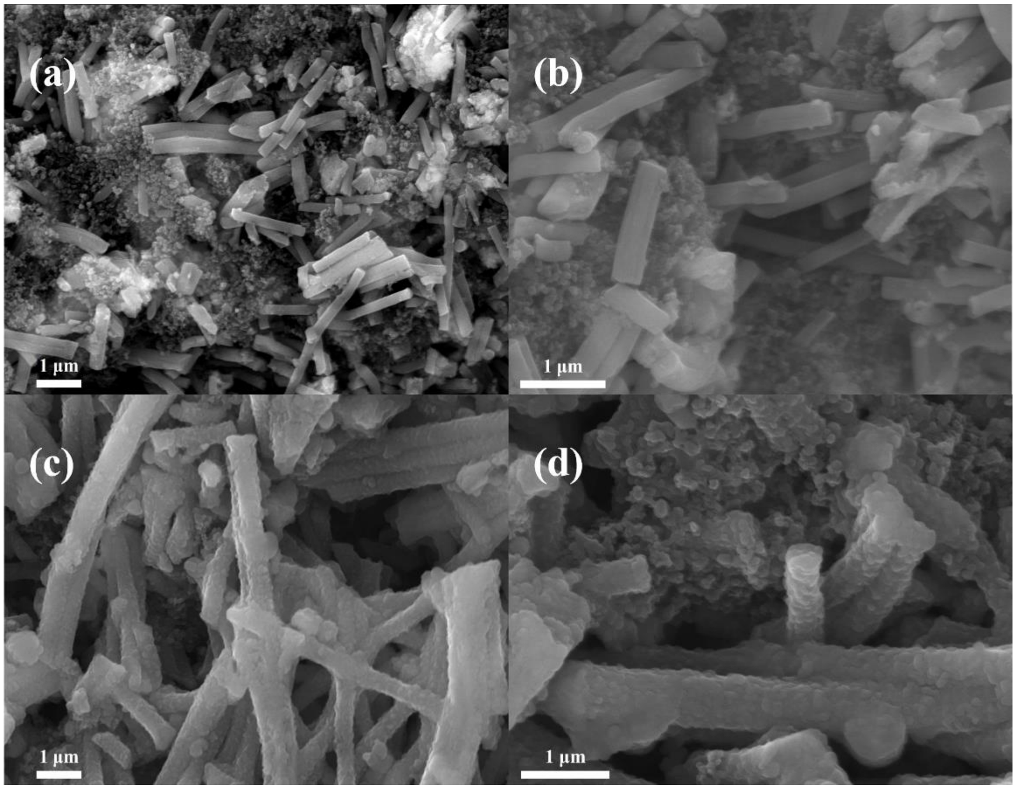

Supplementary Figure 10. (a) and (b) SEM images of Se/Mo@CNFs before cycle at low and high magnification. (c) and (d) SEM images of Se/Mo@CNFs after 1000 cycle at 5C at low and high magnification.

Supplementary Table 1. Elemental analysis of Mo@CNFs.

Element	Weight %	Atomic %	Net Int.	Error %
C K	72.21	79.58	4588.24	4.55
N K	12.96	12.25	144.53	14.98
O K	8.89	7.36	227.91	13.39
Mo L	5.94	0.82	374.87	3.91

Supplementary Table 2. Comparison of specific capacity between the present study (Se/Mo@CNFs) and other selenium electrodes in recent reports.

Selenium cathodes	C rate	cycle numbers	Spec. capacity (mAh g^{-1})	Ref.
Se@CoSA-HC	50	5000	267	1
APPC/Se@PDA	1	200	645	2
Ni-NC/Se	4	1000	225	3
Se/Co-NC	1	200	480	4
Se-CMK-3	0.05	100	488.7	5
PNCNFs/Se@MXene	5	5000	348	6
Se/PBC	0.2	200	509	7
Se/Mo@CNFs	1	500	535	This study

References:

1. Tian H, Tian HJ, Wang SJ, et al. High-power lithium-selenium batteries enabled by atomic cobalt electrocatalyst in hollow carbon cathode. *Nat Commun* 2020; 11: 12. Article. DOI: 10.1038/s41467-020-18820-y.
2. Cao YQ, Lei FF, Li YL, et al. A MOF-derived carbon host associated with Fe and Co single atoms for Li-Se batteries. *Journal of Materials Chemistry A* 2021; 9: 16196-16207. Article. DOI: 10.1039/d1ta04529f.
3. Li JY, Jiang JX, Zhou YG, et al. Nickel single-atom catalysts on porous carbon nanosheets for high-performance lithium-selenium batteries. *Energy* 2023; 285: 8. Article. DOI: 10.1016/j.energy.2023.129434.
4. Gao F, Yue XA, Xu XY, et al. A N/Co co-doped three-dimensional porous carbon as cathode host for advanced lithium-selenium batteries. *Rare Metals* 2023; 42: 2670-2678. Article. DOI: 10.1007/s12598-023-02273-5.
5. Zhang Q, Cai LT, Liu GZ, et al. Selenium-Infused Ordered Mesoporous Carbon for Room-Temperature All-Solid-State Lithium-Selenium Batteries with Ultrastable Cyclability. *Acs Applied Materials & Interfaces* 2020; 12: 16541-16547. Article. DOI: 10.1021/acsami.0c01996.
6. Li JY, Song JJ, Luo LQ, et al. Synergy of MXene with Se Infiltrated Porous N-Doped Carbon Nanofibers as Janus Electrodes for High-Performance Sodium/Lithium-Selenium Batteries. *Advanced Energy Materials* 2022; 12: 12. Article. DOI: 10.1002/aenm.202200894.
7. Ma CH, Wang HL, Zhao XS, et al. Porous Bamboo-Derived Carbon as Selenium Host for Advanced Lithium/Sodium-Selenium Batteries. *Energy Technology* 2020; 8: 8. Article. DOI: 10.1002/ente.201901445.

Supplementary Figure 1. The figure legends are required to have the same font as the main text, 12-point normal Times New Roman, single spaced. Please use a single paragraph for each legend and prepare the figures keeping in mind the PDF layout.
